# Phenology and Dispersal of the Wheat Stem Sawfly (Hymenoptera: Cephidae) Into Winter Wheat Fields in Nebraska

**DOI:** 10.1093/jee/toaa093

**Published:** 2020-05-25

**Authors:** Chris T McCullough, Gary L Hein, Jeffrey D Bradshaw

**Affiliations:** 1 School of Plant and Environmental Sciences, Virginia Tech, Blacksburg, VA; 2 Department of Entomology, University of Nebraska-Lincoln, Lincoln, NE; 3 Panhandle Research and Extension Center, University of Nebraska-Lincoln, Scottsbluff, NE

**Keywords:** ecology, pest management, movement, Nebraska, wheat

## Abstract

Historically, the wheat stem sawfly, *Cephus cinctus* Norton was a pest in spring wheat-growing regions of the northern Great Plains. However, in the 1980s, it was found infesting winter wheat fields in Montana. Infestations were first detected in western Nebraska in the 1990s, and have since spread throughout the Nebraska Panhandle. Larval damage occurs from stem-mining, but stem girdling that results in lodged stems that are not harvested results in the greatest yield losses. The biology and phenology of the wheat stem sawfly are well described in the northern portion of its range, but they are lacking in Colorado, southeast Wyoming, and Nebraska. In this study, the phenology and dispersal of the wheat stem sawfly in Nebraska winter wheat fields is described using sweep net and larval sampling. During this 2-yr study, adult activity began on May 23 and ended on June 21. Adult sex ratios were 2.32 males per female in 2014 and 0.46 males per female in 2015. Both sexes demonstrated an edge effect within the wheat fields, with greater densities near the field edge. The edge effect was stronger for male wheat stem sawfly than females. Wheat stem sawfly larval density also had an edge effect, regardless of the density of female wheat stem sawfly present. This information will be useful for developing management plans for the wheat stem sawfly in Nebraska and neighboring regions.

The wheat stem sawfly, *Cephus cinctus* Norton is a significant insect pest of wheat, *Triticum aestivum* L. (Cyperales: Poaceae), in the northern Great Plains. The wheat stem sawfly causes an estimated $350 million in losses each year in the northern Great Plains (Beres et al. 2011). It was first documented infesting spring wheat near Souris, Manitoba in 1895 (Ainslie 1920). Throughout most of the 20th century, winter wheat in the Great Plains escaped wheat stem sawfly damage as the wheat stem sawfly was not synchronized with winter wheat phenology (Lou et al. 1998). By the mid-1980s, wheat stem sawfly populations had synchronized their life cycle with winter wheat, rendering it vulnerable to infestation as well (Morrill and Kushnak 1996). This adaptation coincided with increased reports of economic wheat stem sawfly infestations occurring farther south than previously reported. The wheat stem sawfly now affects the winter wheat growing areas of southeastern Wyoming, the Nebraska Panhandle, and northeastern Colorado (Bradshaw and Hein, unpublished data; Irell and Peairs 2014).

Upon emergence in the spring, female wheat stem sawfly disperse in search of hosts. The lifespan of an adult wheat stem sawfly is about 1 wk (Wallace and McNeal 1966). In Nebraska, the wheat stem sawfly flight period lasts from mid-May to late June (McCullough 2016). Wheat stem sawfly reproduces via arrhenotokous parthenogenesis (Mackay 1955). Females prefer to oviposit fertilized eggs in larger stems (Cárcamo et al. 2005). After hatching, larvae feed on the vascular tissue within the stem (Delaney et al. 2010). The first larva to hatch typically consumes conspecifics within the same stem resulting in only one larva surviving per stem (Ainslie 1920). Larvae continue feeding until an increase in light penetration of the stem wall, and a drop in plant moisture signal them to move down the stem to prepare for diapause (Holmes 1975). Above the diapause site, the larva will girdle the inside of the stem and plug the girdled end with frass. Through the winter, larvae must accumulate sufficient cooling days to terminate diapause and resume development (Salt 1947). After development resumes, pupation occurs and after about 3 wk adult emergence begins (Perez-Mendoza and Weaver 2006a).

Wheat stem sawfly larvae limit yield potential through two mechanisms. Larval stem mining interferes with nutrient transfer to the grain, resulting in reduced grain size and a 10–20% physiological reduction in yield (Delaney et al. 2010). Additionally, the stem girdling behavior of the wheat stem sawfly weakens the stem, making it prone to lodging. Lodged wheat stems are difficult for typical combine harvest operations to recover. The amount of stem lodging can vary with weather conditions leading up to harvest, as an external force is needed to lodge the stem.

Because of the damage potential, management tactics are directed against the larvae. Planting of solid stem wheat varieties is the primary management tactic used against the wheat stem sawfly. Solid stem varieties crush wheat stem sawfly eggs, increase desiccation, inhibit larval movement, and result in less fecund females emerging (Holmes and Peterson 1961, 1962; Cárcamo et al. 2005). In the absence of wheat stem sawfly, yields of solid stem varieties have been shown to be 10–15% less than hollow stem varieties; therefore, solid stem varieties are most economical when grown in fields with medium to heavy wheat stem sawfly pressure (Berg et al. 2015). Additionally, pith expression for solid stem wheats is positively correlated to the number of sunny days during the early growing season and precipitation later in growing season, leading to variable pith expression between years and locations for solid stem varieties (Holmes 1984, Beres et al. 2017). Border modifications or the use of seed blends to limit the amount of solid stem wheat planted have had some success in limiting wheat stem sawfly damage; however, planting the entire crop to a solid stem variety remains the best option (Beres et al. 2009). Recent molecular investigations into barley’s resistance to wheat stem sawfly larvae and the characterization of wheat’s response to wheat stem sawfly larval feeding have the potential to create new mechanisms of plant resistance against the wheat stem sawfly (Biyiklioglu et al. 2018, Varella et al. 2018).

Higher population densities near the edge of wheat fields nearest the previous year’s wheat field (i.e., wheat stem sawfly source) have been observed for all life stages of the wheat stem sawfly in Montana (Morrill et al. 2001, Weaver et al. 2004, Nansen et al. 2005a). This edge effect continued to decline up to 90 m into the field (Goosey 1999, Morrill et al. 2001, Weaver et al. 2004). These studies often combined male and female wheat stem sawfly for analysis. It may be practical to present wheat stem sawfly densities in aggregate, but it is the females that are ovipositing in wheat stems. Goosey (1999) and Sing (2002) analyzed wheat stem sawfly dispersal into wheat separately for each sex. Sing (2002) found that female densities do not decline as dramatically as male densities farther into the wheat field.

The distribution of wheat stem sawfly eggs and larvae also show an edge effect with the greatest densities closest to the emergence source for the adults (Nansen et al. 2005a). This edge effect for wheat stem sawfly larvae is not consistent, as Sing (2002) only detected it in three of nine fields sampled. However, inferences about adult dispersal based on the distribution of eggs and larvae may inaccurately represent the movement of adults as only one larva survives per stem. Multiple eggs or larvae can be found within a stem, but without further testing, it is unknown if the eggs came from different wheat stem sawfly (Nansen et al. 2005a). Additionally, the dispersal of males is unlikely to be related to egg or larval densities. Clarification of these differences between adult and larval wheat stem sawfly would enable more informed management decisions.

It is also important to understand the phenology and ecology of the wheat stem sawfly in the southern portion of its range where economic infestations are more recent. The wheat stem sawfly is well studied in the northern Great Plains. However, the phenology of the wheat stem sawfly is likely different in these new areas where it is emerging as a pest. Pest managers need more regionally specific details about the phenology of the wheat stem sawfly in these areas. The objectives of this study were to describe the phenology of the adult wheat stem sawfly activity in western Nebraska winter wheat fields and to determine the activity levels of adult wheat stem sawfly as they disperse into wheat fields adjoining their emergence sites.

## Materials and Methods

### Field Locations

Three commercial dryland winter wheat fields in the Nebraska Panhandle were each sampled in 2014 and 2015. Each field utilized a wheat-fallow rotation. These fields were located near the towns of McGrew (41.701264° N, −103.464840° W) (elevation 1,184 m), Gurley (41.312453° N, −103.019170° W) (elevation 1,314 m), and Hemingford, NE (42.288374° N, −103.139229° W) (elevation 1,320 m). The wheat variety ‘Goodstreak’ was grown at McGrew both years. At the McGrew field, wheat and fallow were alternated between 80 m wide × 500 m long tracts. Two of these tracts were sampled each year. Tillage was used on the fallow tract with a tandem disc in the spring of 2014. No tillage was used on the fallow tract in 2015 due to wet field conditions. The soil type at this location was Bridget. Goodstreak was grown at Gurley in 2014, and ‘Settler CL’ was used in 2015. Wheat and fallow were rotated between two, 420 m wide × 800 m long tracts. Weeds in the fallow tract were managed with herbicides. Kuma, Duroc, Satanta, and Alliance soil types were present in this field. Settler CL was grown both years at Hemingford. The wheat sampled was in two different sized tracts. In 2014, the tract was 200 × 800 m, and in 2015 it was 330 × 800 m. Weeds in the fallow tract were managed using herbicides. Duroc and Alliance were the soil types at this location.

### Sampling Methods

**Fig. 1.**
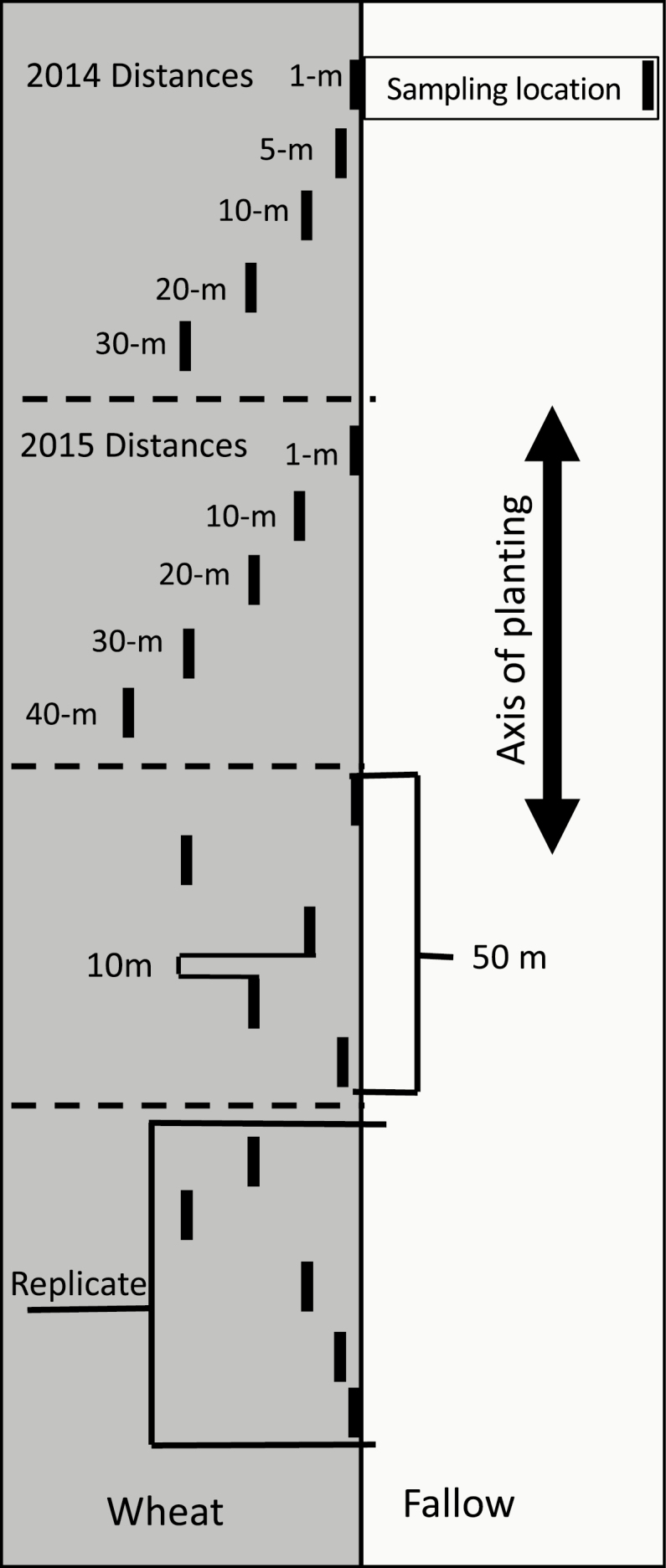
Diagram of sampling scheme. The order that sampling distances were placed along the axis of planting was randomized within each replicate.

To sample adult wheat stem sawfly in 2014, sweep net samples were taken at 1, 5, 10, 20, and 30 m into the wheat from the field edge (Fig. 1). In 2015, sweep net samples were taken at 1, 10, 20, 30, and 40 m into the wheat from the field edge (Fig. 1). Sample locations were spaced 10 m apart along the axis of planting, and together, these five sampling distances made up a replicate extending 50 m along the edge of the field. The order of sampling of each distance was randomized within each replicate. Replicates were repeated as many times as allowed by the field dimensions. In 2014, there were 18 replicates at McGrew, 14 at Gurley, and 16 at Hemingford. In 2015, McGrew had 16 replicates, Gurley had 15, and Hemingford had 16.

An individual sample consisted of 20 sweeps that were taken while walking parallel to the direction of the row and sweeping through the upper third of the wheat canopy with a 38-cm diameter sweep net. All sampling was conducted by one person between 0900 and 1500 hours each day. The sampler started at a location marker and continued along the row, covering an area of about 13 m^2^. A 180° arc across the rows was considered one sweep. Biweekly sampling began in early May and continued through June, when wheat stem sawfly were no longer present. All wheat stem sawfly samples were bagged and returned to the lab for counting and sex determination.

In 2015, wheat stem sawfly larvae were sampled from within the sweep sample areas. Within each sample location, two noncontiguous wheat stem samples were randomly selected following the adult flight period and before harvest. Each stem sample consisted of all the wheat stems in 50 cm of a single wheat row. All stems from these samples were split to check for larval presence. Visual confirmation of a larva, a larval cadaver, pupation chamber of a *Bracon* sp. (wheat stem sawfly parasitoid), or larval frass trail counted as larval presence.

### Statistical Analysis

An analysis of variance was performed using PROC GLIMMIX (SAS 2013a) to test the main effects and interaction of the sex of the wheat stem sawfly and distance from the edge on adult wheat stem sawfly density with sampling date as a repeated measure. A negative binomial distribution was fit to these data. Replicate at each field was treated as a random effect. The autoregressive-one covariance structure provided the best fit to the data as it had the lowest Akaike’s Information Criterion value of the tested covariance structures. Mean comparisons were evaluated using Tukey’s Honestly Significant Differences. Separate analyses were done for each field for each year. Dates used for this analysis were limited to dates when 15 or more wheat stem sawfly were caught to improve model performance.

An analysis of covariance was used (PROC GLIMMIX) to test for changes in larval density over sampling distances. The number of stems in each sample was moderately correlated (*r* = 0.53) to larval density, thus stem density was included as a covariate of distance. Replicates were included as a random effect. These data were not normally distributed and a negative binomial distribution was fit to them. Tukey’s Honestly Significant Differences were used for mean comparisons of the number of larvae found between sampling distances. Each field was analyzed separately.

Wheat stem sawfly seasonality was characterized by calculating the proportional accumulation of wheat stem sawfly sampled by date. To provide uniformity among sampling dates, dates were represented by the growth stage of the wheat at each field using the Zadoks Decimal scale (Zadoks et al. 1974). Zadoks growth stage was approximated by visual assessment. A repeated-measures analysis of variance using PROC MIXED (SAS 2013b) was used to test for differences in the rate of accumulation between the sexes of wheat stem sawfly. Location and year were treated as random effects. The autoregressive-one covariance structure provided the best fit to the data according to Akaike’s Information Criterion. Tukey’s Honestly Significant Differences were used for mean comparisons.

## Results

**Fig. 2.**
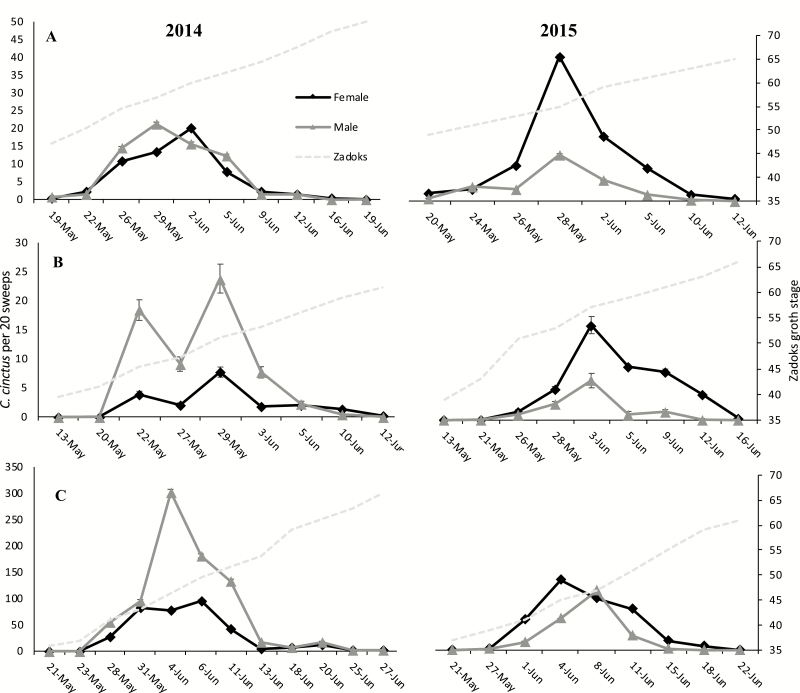
Mean ± SEM of wheat stem sawfly adults sampled per 20 sweeps within winter wheat near McGrew (A), Gurley (B), and Hemingford (C), NE in 2014 and 2015 throughout the flight period.

In 2014, the first wheat stem sawfly were sweep net sampled on May 14, and the last wheat stem sawfly were sampled on June 27. In 2015, the first wheat stem sawfly were sampled on May 12, and the last wheat stem sawfly were caught on June 25. The first adults sampled each year were seen at McGrew followed by Gurley then Hemingford (Fig. 2). Each year, the greatest densities of wheat stem sawfly occurred at the end of May and beginning of June. This timing coincided with wheat head emergence, Zadoks 51–59. The end of the flight period followed the same order between locations as emergence. A total of 26,428 adult wheat stem sawfly were sampled in 2014 with a ratio of 2.32 males per female. In 2015, 16,623 adult wheat stem sawfly were sampled with a ratio of 0.46 males per female. Of the total number of adult wheat stem sawfly sampled in 2014 and 2015, 70 and 63%, were caught at Hemingford each year, respectively.

**Fig. 3.**
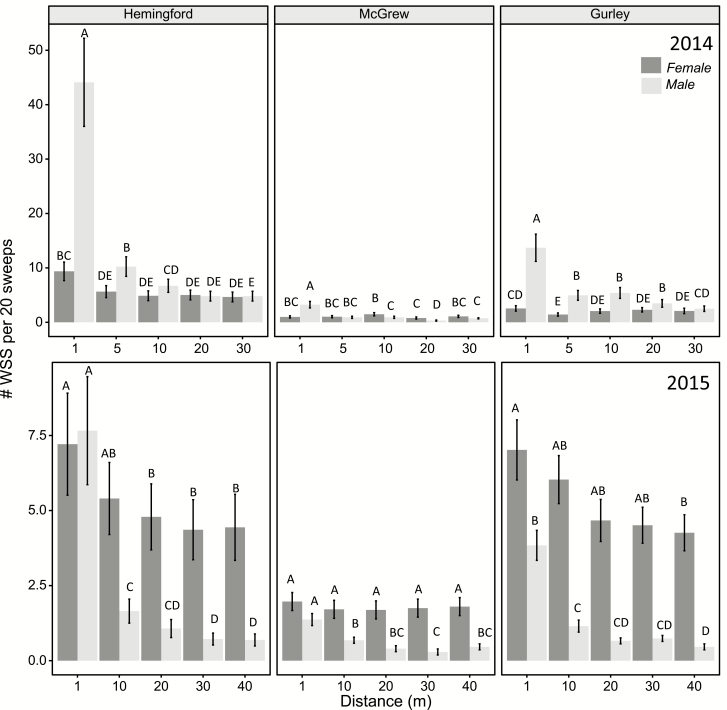
The average number of adult wheat stem sawfly sampled at various distances into winter wheat fields in 2014 and 2015. Means followed by the same letter are not significantly different (*P* > 0.05) (Tukey’s Honestly Significant Difference).

A significant sex by sampling distance interaction was detected for sweep sampled wheat stem sawfly at Gurley (*F*_4,65_ = 27.8, *P* < 0.0001), Hemingford (*F*_4, 75_= 42.7 *P* < 0.0001), and McGrew (*F*_4, 85_ = 29.2, *P* < 0.0001) during the 2014 flight period. These interactions also were significant in 2015 at Gurley (*F*_4, 70_ = 22.9, *P* < 0.0001), Hemingford (*F*_4, 75_ = 61.9, *P* < 0.0001), and McGrew (*F*_4, 75_ = 12.5, *P* < 0.0001). The highest densities of male wheat stem sawfly were found at the 1 m distance with densities decreasing with increasing distance into the field (Fig. 3). Female densities were only greatest at the 1 m distance at Hemingford in 2014. Otherwise, distances farther into the field had similar female wheat stem sawfly densities as the 1 m distance. For McGrew in 2015, female sawfly density did not change with distance. Overall, in 2014, 46–56% of males and 19–33% of females were caught at the 1 m distance. In 2014, 8–12% of all males and 15–20% of all females were caught from the 30 m distance. In 2015, 41–67% of males and 20–28% of females were sampled from the 1 m distance. At the 30 m distance in 2015, 5–9% of males and 16–22% of females were caught.

Larval and stem density field averages were highest at Hemingford, with 196.6 ± 3.34 (mean ± SEM) stems per row meter and 102 ± 4.8 larvae per row meter (52% infestation). Stem density at Gurley averaged 138.2 ± 2.54 stems per row meter with 68.7 ± 3.0 larvae per row meter (50% infestation). McGrew had the lowest stem and larval density field averages with 121.34 ± 2.20 stems per row meter and 61.0 ± 2.3 larvae per row meter (50% infestation).

**Fig. 4.**
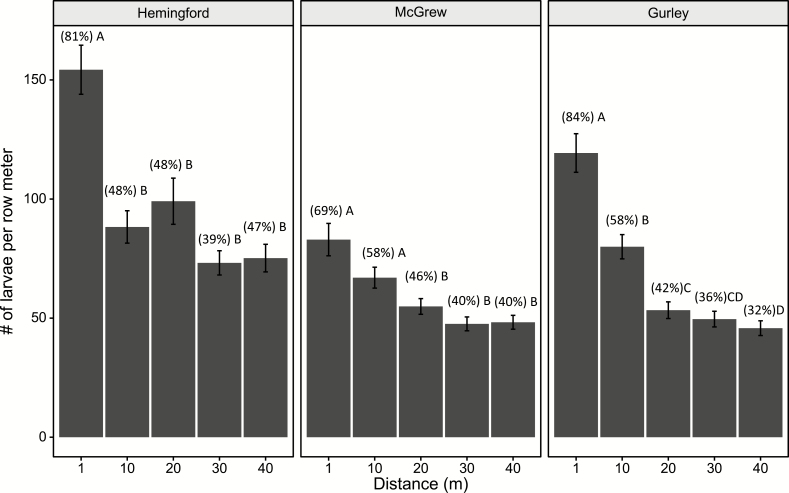
The average number of wheat stem sawfly larvae per row meter sampled at various distances into winter wheat fields in 2015. Numbers in parenthesis are the percentage of stems infested. Means followed by the same letter are not significantly different (*P* > 0.05) (Tukey’s Honestly Significant Difference).

Larval density was highest at the field edge (1 m distance) for each field. Hemingford had the greatest larval density at 1 m, averaging 154.38 ± 10.3 larvae per row meter, (81% infestation). Larval density only significantly declined from the 1 m to the 10 m distance at Hemingford; otherwise, no changes in larval density occurred from between the 10 m and 40 m distances (Fig. 4) McGrew had the lowest larval density at the 1 m distance with 83.0 ± 6.8 larvae per row meter (69% infestation). Larval density at McGrew significantly declined from the 1 m distance at the 20 m distance (Fig. 4). Gurley averaged 119.3 ± 8.1 larvae per row meter (84% infestation), at the 1 m distance. Gurley was the only field to show a continued decline of larval density as the sampling distance increased into the field (Fig. 4) All three fields showed a 60–40% decline in larval density from the 1-m to the 40-m sampling distance (Fig. 4).

**Fig. 5.**
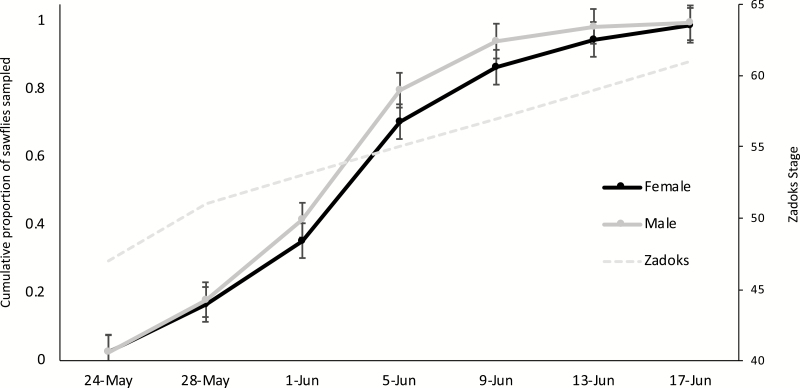
Cumulative mean ± SEM proportion of male and female wheat stem sawfly collected via sweep net samples averaged over six site-years.

For the accumulated wheat stem sawfly activity through the season via sweep sampling, no date × sex interaction was found (*f*_6, 35_ = 1.62, *P* = 0.17). The main effects of sex of the wheat stem sawfly and sampling event for accumulated wheat stem sawfly activity were significant (*f*_1, 35_ = 14.39, *P* = 0.0006; *f*_6, 6_ = 103.18, *P* < 0.0001). Overall, males accumulated earlier than females, with 4 ± 1% more of their sampled population per sampling event (Fig. 5). Male emergence was nearly complete by June 9, with 95% of all males being sampled by then, compared to 85% for females. The largest gains in the populations sampled were from the June 1 to June 5 sampling dates, Zadok’s 53–55. The accumulation for both sexes of wheat stem sawfly increased by 35 and 38% for females and males, respectively.

## Discussion

The adult activity period occurred earlier in Nebraska, May 12–June 27, than is often reported in Canada and Montana. The flight period in Canada is reported to last from June 10 through July 10 (Beres 2011). By the time adult activity begins in Canada, peak adult activity has passed for most of the Nebraska Panhandle. There is some overlap with the activity period in Montana, where the flight period starts around May 25 and can last until the end of July (Morrill and Kushnak 1999). A latitudinal gradient was observed in the timing of the flight period in Montana (Goosey 1999). A similar effect could be inferred with the differences in wheat stem sawfly phenology between the Gurley and Hemingford sites. The earlier wheat stem sawfly activity at McGrew might be due to differences in temperature caused by the differences in elevation.

The greatest densities of adult wheat stem sawfly sampled were recorded at the 1-m sampling distance. With the exception of females sampled at McGrew, sawfly densities decreased farther into the wheat field. The changes in wheat stem sawfly density from the edge to the most interior distance were greater for males than females, a 90 and 60% drop in density, respectively. Densities of male wheat stem sawfly declined up to the 20 m distance. For females, no decline in density was found after 5 m in 2014 and 10 m in 2015. Similar differences in wheat stem sawfly dispersal between the sexes have been observed in Montana wheat fields with male wheat stem sawfly having greater declines than females the farther into the field (Sing 2002).

By aggregating along the edge, males may increase the concentration of 9-acetyloxynonanal, a volatile sex pheromone used by males to attract females, and this would increase the likelihood of males encountering females (Cossé et al. 2002). Aggregation along the field edge also increases the level of competition for females; however, this likely would remain the best chance for males to mate. When considering the wheat stem sawfly in a patchy grassland setting, aggregating to increase their pheromone plume may be advantageous because proximity to emerging females would not be guaranteed. The value of this would be minimized in a wheat-fallow rotation because of the close proximity between the previous year and current year hosts and the density and concentration of these hosts.

Females do not discriminate between infested and uninfested hosts for oviposition (Buteler et al. 2009). Thus, females dispersing farther into the field to oviposit would decrease the chance their offspring will be cannibalized as a result of multiple eggs being laid per stem (Buteler et al. 2015). Sing (2002) found that the dispersal of female wheat stem sawfly was well described as diffusion. The data presented here support this claim. Females wheat stem sawfly are moving from an area of high density at the field edge to an area of lower wheat stem sawfly density farther into the field. All females have to enter the field at the edge, driving up female densities at the 1 m distance. Females seemingly move towards the middle of the field as they are unlikely to move back into the fallow due to an assumed lack of hosts. Within the field, females may be moving back towards the field edge, laterally, or remaining in areas with high host quality. Female wheat stem sawfly may also be responding to the higher density of female wheat stem sawfly near the edge and move to areas with fewer female wheat stem sawfly.

An interesting pattern is the uniformity of females across distances at McGrew in 2015. Each field had varying densities of downy brome, *Bromus tectorum* L., present. Hemingford had no downy brome, Gurley had downy brome near the field edge, but McGrew was heavily infested with downy brome throughout, particularly within the first 10 m of wheat from the field edge. Female sawflies prefer downy brome to wheat as a host (Perez-Mendoza et al. 2006b), so greater densities of females could be expected in these areas; however, this was not observed at these fields. Downy brome can reach densities of 200 plants per row meter and outcompete wheat (Blackshaw 1993). Such dense stands of downy brome can create a sampling environment that is difficult to sweep sample. These conditions occurred at the McGrew field, where downy brome was the dominant plant. Finally, the downy brome in these fields senesced before wheat, reducing the number of viable hosts within some of the sample distances and could have caused females to disperse further into the field in search of suitable hosts.

Even though female densities did not vary with distance from the field edge at McGrew in 2015, larval density declined at the 20 m distance. At Gurley in 2015, female densities only differed between the 1 m distance and the 40 m distance, but larval densities continued to decline up to the 40 m distance. At Hemingford in 2015, female densities declined after the 10 m distance, matching the decline in larval density. Regardless of the female density, the larval density displays an edge effect. Sing (2002) only detected an edge effect for larvae in three of nine sites sampled; but, found that female wheat stem sawfly more reliably had an edge effect. Nansen et al. (2005b) more frequently detected an edge effect for larval wheat stem sawfly but had fields that did not have an edge effect. Females oviposit 50 eggs, on average, over their lifespan (Ainslie 1920), but females will be exposed to thousands of suitable stems for oviposition by the time they are 40 m into a wheat field. Further study of wheat stem sawfly dispersal behavior is needed to clarify the connection between adult female and subsequent larval density, particularly if a sampling plan based on adult wheat stem sawfly is to be developed.

According to Holmes (1982), stem density is predictive of the size of the wheat stem sawfly population potential within a field. However, on a proportional basis at each field, only about half of all the wheat stems sampled for wheat stem sawfly larvae were infested. Holmes (1982) noted that fields with the lowest densities of adult wheat stem sawfly had the greatest infestation potential, in that the reduced intraspecific competition would allow for rapid population growth. However, there is a saturation point with the population where new stems are not being infested, and larval cannibalism is limiting the ability of the wheat stem sawfly population to grow (Holmes 1982). The three fields sampled here illustrate Holmes’ point. The McGrew field had the lowest density of female wheat stem sawfly and wheat stems sampled but had a similar proportion of stems infested as Hemingford and Gurley. The high wheat stem sawfly densities and intraspecific competition at Hemingford is likely limiting the population there, while it is the number of suitable wheat stems that likely limited the population at McGrew. When sampling for wheat stem sawfly, either adults or larvae, it is important to consider stem density, otherwise lower densities of wheat stem sawfly have the potential to be dismissed when developing management plans.

A decrease in adult density was observed as distance increases from a field edge, i.e., a population ‘edge effect’. This effect is much more pronounced for adult male densities than female densities. It is more distinct when adult densities are higher, such as at Hemingford. More than half of the males caught were taken from the 1 m distance compared about 30% for females. Almost 20% of females were caught 30 m into the wheat field compared to only 7% for males. Larval density also showed and edge effect, declining from around 75% of wheat stem infested at the 1 m distance to around to 40% of stems at the 30 m distance. Therefore, taking management actions, like planting solid stem wheat varieties, near field edges may be a plausible tactic; however, the scale of these actions needs to consider the degree that female dispersal and oviposition behavior extends into the wheat. In the fields sampled, wheat head emergence appears to be an ideal time to sample adult wheat stem sawfly in order to maximize the likelihood of detecting them. Further investigation of female wheat stem sawfly dispersal into wheat fields may reveal further spatial relationships between wheat stem sawfly density, host, and subsequent oviposition. The information presented here can be used to better inform pest management decisions, such as timing of sampling for adult wheat stem sawfly, in geographic areas where the wheat stem sawfly has recently increased in pest status.
